# Peritoneal Dialysis and Inflammatory Demyelinating Polyneuropathy: A Correlation or Co-Incidence?

**DOI:** 10.7759/cureus.27095

**Published:** 2022-07-21

**Authors:** Kunal Bhuta, Andres Cordova Sanchez, Ayorinde Soipe, Haris Mobeen, Kriti Devkota

**Affiliations:** 1 Department of Internal Medicine, State University of New York (SUNY) Upstate Medical University, Syracuse, USA

**Keywords:** electromyography, emg, peritoneal fluid, diabetes mellitus, kidney failure, chronic kidney failure, uremic neuropathy, end stage renal disease, chronic inflammatory demyelinating polyneuropathy, peritoneal dialysis

## Abstract

Uremic neuropathy (UN) is a sensorimotor polyneuropathy typically affecting the lower extremities due to length-dependent demyelination and axonal degeneration. Hemodialysis (HD) and peritoneal dialysis (PD) are the two widely used modalities for treating end-stage renal disease (ESRD) patients. Today, with the understanding of solute and water kinetics, PD is considered equivalent to in-center HD. Chronic inflammatory demyelinating polyneuropathy (CIDP) manifests as symmetric, motor-predominant neuropathy that results in both proximal and distal muscle weakness. It is treatable with immune modulatory therapies.

Here, we present a series of three patients who developed CIDP following the initiation of PD. Patient A: 39-year-old male with ESRD secondary to renal dysplasia presented with new onset neuropathy four months after starting PD. Patient B: 30-year-old male with ESRD secondary to IgA nephropathy presented with a history of numbness in his feet gradually progressing to his legs 12 months after initiating PD. Patient C: 56-year-old female with ESRD and uncontrolled diabetes mellitus presented with progressive muscle weakness four months after initiating PD. These three patients were all on continuous cycling PD. They were followed at three different dialysis units and were initiated on CCPD at different times. All of these patients were found to have CIDP on electromyography. Patients A and B were treated with IV immunoglobulin (IVIG) and improved, while patient C received plasmapheresis and improved. It has been recognized that PD solution is not physiological and may lead to activation of the host immune system triggering an autoimmune demyelinating process. Immunologic pathogenesis is not clearly understood. Macrophage activation and cytokines may play a role in the demyelination process. With the recent initiative to increase the use of PD, more studies are warranted to understand this uncommon complication.

## Introduction

Peripheral neuropathy is a common neurologic problem. It is a disorder that affects the cell body, axon, or myelin of motor or peripheral sensory neurons and can be classified as neuropathological, axonal, or demyelinating. While it can be seen due to various causes, it is also seen in 60-100% of patients on dialysis [1]. It is a sensorimotor polyneuropathy that typically affects lower extremities due to length-dependent axonal degeneration [2]. Uremic neuropathy (UN) is thought to be secondary to oxidative stress-related nerve injury from an accumulation of uremic toxins. Although the exact mechanism remains unknown, it is hypothesized that hyperkalemia and hyperphosphatemia may contribute to the development of uremic encephalopathy [3]. With improvements in different dialysis modalities, the incidence of UN has reduced. Both hemodialysis (HD) and peritoneal dialysis (PD) have been shown to be effective modes of delivering dialysis to patients with end-stage renal disease (ESRD). The first attempts to use a peritoneal membrane to dialyze uremic toxins were made almost 100 years ago. In 1850, eminent Scottish chemist, Thomas Graham, demonstrated that membranes could be semipermeable [4]. Understanding solute and water kinetics allowed for the successful application of PD to patients with acute kidney injury (AKI) and ESRD. The first human PD was attempted in the year 1923. Since then, PD techniques have undergone significant evolution paving the way for improved and more efficient techniques for PD. Today, PD is considered equivalent to in-center HD [5]. However, PD is not without its complications. Here we would like to present a case series of three patients admitted to the university hospital for inflammatory demyelinating polyneuropathy after initiating PD.

## Case presentation

Case 1

A 39-year-old patient with ESRD secondary to renal dysplasia received a living-related donor kidney transplant from his father at the age of 16 years. After losing allograft function, he was started on HD and switched to continuous cyclic peritoneal dialysis (CCPD) in five months. The patient had bilateral lower extremity neuropathy for about four months after starting CCPD, which, at the time, was attributed to uremia and treated with gabapentin. It started with some tingling in his toes, followed by numbness and then weakness which progressed further over six months to neuropathy in his upper extremities. Electromyography (EMG) studies were positive for moderate-to-severe (distal greater than proximal) acquired demyelinating sensory and motor neuropathy with signs of reinnervation and active ongoing denervation. The presence of conduction block in more than two nerves, f-wave slowing and conduction velocity slowing, were consistent with a diagnosis of chronic inflammatory demyelinating polyneuropathy (CIDP). He was eventually admitted to the hospital for a five-day course of IV immunoglobulin (IVIG) therapy. He continued on PD during this time. Post-discharge, he continued to receive IVIG for about six months, and his symptoms improved.

Case 2

A 30-year-old male patient with ESRD secondary to IgA nephropathy on PD presented with tingling and numbness in bilateral lower extremities for three months. It was also associated with slow, progressive, and ascending weakness in bilateral lower limbs. He was started on oral ropinirole every night with partial relief of his symptoms. The patient had noticed these neurological symptoms for the first time about 12 months after starting CCPD. EMG studies showed subacute sensorimotor neuropathy with a combination of demyelinating and axonal features involving both the arms and the legs and nerve conductions showing both sensory and motor involvement, with demyelinating features (reduced conduction velocity, conduction block, and prolonged latencies). He completed IVIG treatment over five days after the infectious workup was negative. He reported a partial improvement in his lower extremity weakness following IVIG. The patient's pain appeared to be better controlled on amitriptyline 37.5 mg nightly, in combination with his gabapentin. He continues to receive IVIG two days a month at present. 

Case 3

A 56-year-old female with ESRD and uncontrolled diabetes mellitus (HbA1c >14%) presented with six months of progressive muscle weakness which started approximately four months after initiating CCPD. The patient started to have paresthesia in her toes and hands. EMG studies showed CIDP. She received IVIG for five days but reported no improvement. She received a second course of IVIG but could not tolerate the fourth dose, and treatment was discontinued. Her symptoms continued to progress. Repeat EMG studies showed demyelinating polyneuropathy with severe secondary axonal loss. She received five days of plasmapheresis inpatient and improved significantly. All of these patients were followed at different dialysis units and were initiated on CCPD at different times.

A summary and comparison of the clinical presentations and demographics of the three patients are presented in Table 1.

**Table 1:** Clinical summary of the three patients. CCPD: Continuous cyclic peritoneal dialysis; CIDP: Chronic inflammatory demyelinating polyneuropathy; IVIG: Intravenous immunoglobulin.

Patient	1	2	3
Age	39	30	56
Sex	Male	Male	Female
Cause of renal failure	Renal dysplasia	IgA nephropathy	Diabetic nephropathy
Duration between initiation of CCPD and CIDP	4 months	12 months	4 months
Worst state	Bed bound	Bed bound	Chair bound
Type of therapy	IVIG	IVIG	IVIG x2 and plasmapheresis
Improvement	Yes	Yes	Yes

## Discussion

CIDP is a demyelinating sensorimotor polyneuropathy which can be subacute or chronic in onset, with elevated cerebrospinal fluid protein levels and nerve conduction abnormalities on electrophysiological studies [6]. Here, we describe a series of three patients who developed progressive demyelinating neuropathy within 4-12 months of initiating CCPD. There was no single cause that could account for this occurrence. Screening tests for autoimmune diseases, vitamin deficiencies, paraproteinemia, and heavy metal poisoning were all negative in all three patients. There was no history of recent exposure to neurotoxic drugs. All patients presented in this report came from different dialysis units using dialysis fluids of different types and companies, which ruled out bio-incompatibility of special dialysis or equipment production series as the cause of their neuropathy. 

UN has been known to run a rapid course and tends to improve with an intensification of the dialysis regime. The adequate response was obtained by altering the PD prescription but it did not help alleviate the symptoms. There have been a few case reports suggesting the temporal relationship between the development of CIPD and the initiation of PD as early as 1998 [7-8]. While in these reports (Table 2), patients developed their symptoms as early as 4-12 weeks, our patients developed their symptoms anywhere between 4 and 12 months. Most case reports show patients undergoing continuous ambulatory peritoneal dialysis (CAPD), while patients in our case series underwent CCPD. It remains unanswered if this difference in the mode of delivering dialysis is what may have led to a later onset of neurological symptoms in our patients compared to other reports. 

**Table 2:** Characteristics of six patients previously reported with progressive demyelinating neuropathy after initiation of continuous ambulatory peritoneal dialysis.

Patient number	1	2	3	4	5	6
Sex/age (years)	Male/45 years	Male/27 years	Male/48 years	Male/41 years	Male/29 years	Male/24 years
Cause of renal failure	Diabetes Mellitus	Ornellanus syndrome	Diabetes Mellitus	Diabetes Mellitus	IgA nephropathy	Unknown
Interval between initiation of Continuous Ambulatory Peritoneal Dialysis and onset of symptoms (weeks)	4 weeks	12 weeks	6 weeks	12 weeks	10 weeks	6 weeks
Demyelinating changes on electrophysiological testing	Yes	Yes	Yes	Yes	Yes	Yes
Response to intensification of dialysis regime	No	Unknown	Unknown	Unknown	No	No
Outcome	Improved	Recovered	Improved	Improved	Recovered	Improved
Reference	Toepfer M et al. (1998) [7]	Toepfer M et al. (1998) [7]	Toepfer M et al. (1998) [7]	Chen J and Guest S (1998) [8]	Lui SL et al. (2003) [9]	Lui SL et al. (2003) [9]

The development of eosinophilic peritonitis has been a known complication of PD and usually responds to oral corticosteroids. This raises the question if CIPD and eosinophilic peritonitis would fall under a spectrum of allergic reactions. Also, it has been recognized that PD solution is unphysiological and, in a susceptible individual, may lead to activation of the host immune system triggering and autoimmune demyelination process [9]. The exact immunologic pathogenesis is not clearly understood. It has been shown that macrophage activation and cytokines, including tumor necrosis factor (TNF), may play a role in the demyelination process [10,11]. One of the studies hypothesized that exposure to PD fluid per se might cause an alteration in the host immune system leading to macrophage and T-cell activation and release of the pro-inflammatory cytokine, including TNF-alpha [12]. Also, Lui SL et al. demonstrated lymphocytic infiltration in sural biopsy specimens and clinical improvement of neuropathy after immunosuppressive therapy and renal transplantation in one of their patients, which may further support this hypothesis [9]. Chen J and Guest S had some success in halting relapses of CIPD after conversion to HD [8].

To the best of our knowledge, there have been only six patients reported in the literature at the time of this report. Patient 3 in this report is the only female patient to be reported thus far. She improved after multiple attempts at IVIG, eventually requiring plasmapheresis, which questions if this clinical entity, when manifests in female patients, is more severe than others.

## Conclusions

In summary, the temporal relationship between CIDP and initiation of PD and improvement with immunomodulatory therapy suggests that it may be a distinct identity. However, at this stage, it continues to remain speculative. The autoimmune-mediated mechanism triggering this process remains to be understood completely. No optimal treatment has been established to treat CIDP developing in patients after initiation of PD. The number of diagnosed cases may be significantly reduced due to the challenge of diagnosing CIDP and its overlap with uremic and diabetic neuropathy. Could CIDP trigger an underlying immune-mediated process, or is polyneuropathy just an extension of the ESRD cascade? Many questions remain to be answered, including the male predominance, incidence, and immunopathogenesis of this condition. With the recent administrative initiative to increase the utilization of PD, more studies are warranted, now more than ever, to fully understand this uncommon complication.
